# Decreased stability of erythroblastic islands in integrin β3-deficient mice

**DOI:** 10.1002/phy2.18

**Published:** 2013-06-28

**Authors:** Zhenghui Wang, Olga Vogel, Gisela Kuhn, Max Gassmann, Johannes Vogel

**Affiliations:** 1 Institute of Veterinary Physiology, Vetsuisse Faculty University of Zürich and Zürich Center for Integrative Human Physiology (ZIHP)Zürich, Switzerland; 2 Institute for Biomechanics, Swiss Federal Institute of TechnologyZürich, Switzerland; 3 Universidad Peruana Cayetano Heredia (UPCH)Lima, Peru

**Keywords:** Bone marrow niche, calnexin, chaperone, erythroblast differentiation, erythroblastic island, integrins, stress erythropoiesis

## Abstract

Erythroblasts proliferate and differentiate in hematopoietic organs within erythroblastic islands (EI) composed of erythropoietic progenitor cells attached to a central macrophage. This cellular interaction crucially involves the erythroid intercellular adhesion molecule-4 (ICAM-4) and αv integrin. Because integrins are biologically active as α/β heterodimers, we asked whether β3 could be a heterodimerization partner of αv integrin in EIs. To this end we compared stress erythropoiesis driven by two different mechanisms, namely that of integrin β3-deficient (β3^−/−^) mice that exhibit impaired hemostasis due to platelet dysfunction with that of systemically erythropoietin-overexpressing (tg6) mice. While compared to the respective wild type (wt) controls β3^−/−^ mice had much less erythropoietic stimulation than tg6 mice β3^−/−^ blood contained more erythrocytes of a lower maturity stage. Unexpectedly, membranes of peripheral erythrocytes from β3^−/−^ mice (but not those from either wt control or from tg6 mice) contained calnexin, a chaperone that is normally completely lost during terminal differentiation of reticulocytes prior to their release into the circulation. In contrast to erythropoietin-overexpressing mice, the erythropoietic subpopulations representing orthochromatic erythroblasts and premature reticulocytes as well as the number of cells per EI were reduced in β3^−/−^ bone marrow. In conclusion, absence of integrin β3 impairs adhesion of the latest erythroid developmental stage to the central macrophage of EIs resulting in preterm release of abnormally immature erythrocytes into the circulation.

## Introduction

Proliferating and differentiating erythroblasts require a specialized microenvironment termed erythroblastic island (EI) (Mohandas and Prenant 1978; Allen and Dexter 1982; Hanspal and Hanspal 1994), normally (for review see, e.g., An and Mohandas 2011; Manwani and Bieker 2008; Mohandas and Chasis 2010) composed of a F4/80 expressing central macrophage surrounded by about 5–30 cells from nucleated proerythroblasts (ProE) through multilobulated reticulocytes (Lee et al. 1988). During differentiation orthochromatic erythroblasts extrude their nucleus that is subsequently engulfed by the central macrophage, a mechanism crucially requiring macrophage – erythroblast interactions (Mohandas and Chasis 2010). Moreover, this interaction enhances proliferation of erythroblasts by speeding up the G0/G1 phase to complete more cell divisions per time unit and, thus, generating more reticulocytes (Hanspal and Hanspal 1994). In addition, erythroblast–erythroblast interactions regulate survival of erythroblasts through Fas–FasL interaction (Liu et al. 2006). In man the direct contact between erythroblasts is also essential for homotypic signaling between erythroblasts within the EI niche as a mechanism for regulating GATA-1 activity to complete terminal differentiation (De Maria et al. 1999).

The above-mentioned interactions are mediated by a diverse array of adhesion molecules. Erythroblast macrophage protein (Emp) was the first molecule described to be involved in the attachment of erythroblasts to the central macrophage and to neighboring erythroblasts (Hanspal and Hanspal 1994). Subsequently α4β1 integrin in erythroblasts and vascular cell adhesion molecule 1 (VCAM-1) in central macrophages (Sadahira et al. 1995) and erythroid intercellular adhesion molecule-4 (ICAM-4, also known as the Landsteiner and Weiner (LW) blood group glycoprotein (Bailly et al. 1994)) as well as macrophage αv integrin was found to contribute to the erythroblast – macrophage interactions (Lee et al. 2006).

As it has been shown that αIIbβ3 and αvβ3 integrins can interact with ICAM-4 on red blood cells (RBCs) (Hermand et al. 2003, 2004), the present study aimed to compare characteristics of stress erythropoiesis driven by two different mechanisms, for example, in integrin β3-deficient mice that resemble human Glanzmann thrombasthenia characterized by mild gastrointestinal and cutaneous hemorrhage due to impaired platelet aggregation (Hodivala-Dilke et al. 1999) with that of Epo-overexpressing mice (tg6) having independent of oxygen tension about 10-times elevated Epo plasma levels and hematocrit values of 0.8–0.9 (Ruschitzka et al. 2000). Despite having less stimulated erythropoiesis than tg6 mice, peripheral blood of β3-deficient mice contained RBCs of a lower maturation stage. Surprisingly, erythrocytes from β3^−/−^, but not from tg6 mice, contained calnexin, an endoplasmatic reticulum (ER) glycoprotein chaperone that is normally completely lost during terminal RBC differentiation (Patterson et al. 2009). Moreover, the erythropoietic subpopulation representing orthochromatic erythroblasts and premature reticulocytes as well as the number of cells per EI was reduced in β3^−/−^ bone marrow. These findings suggest that β3 integrin could be involved in attachment of late developmental stages of RBCs to the EI.

## Materials and Methods

### Animals

Mice used for the present study were generated previously and genotyped as described (Hodivala-Dilke et al. 1999; Ruschitzka et al. 2000). Integrin β3^−/−^ mice were bred on a 129Sv D3 background and tg6 mice on a C57BL6 background. All mice were aged between 4 and 6 months and the experiments conformed governmental and institutional guidelines.

### Analysis of peripheral blood

Hematological parameters were determined with standard techniques. Plasma erythropoietin concentrations were measured with EPO-Trac™ ^125^I RIA kit (DiaSorin, Stillwater, MN) and RBC osmotic fragility, flexibility, and life span as described (Vogel et al. 2003; Bogdanova et al. 2007). RBC membrane proteins analysis (Bogdanova et al. 2007) revealed for β3^−/−^ mice an additional band that was analyzed by mass spectrometry (Functional Genomics Center Zürich, Switzerland). Optical density ratios between bands 4.1a and 4.1b and the extra band and band 4.2 were determined as described (Gassmann et al. 2009). RBCs were also analyzed flow cytometrically (cf. below) for binding of FITC-labeled Annexin (apoptosis detection kit, PromoKine, Heidelberg, Germany), reticulocyte number (Retic-COUNT, BD Biosciences, Allschwil, Switzerland), remnants of ER (ER-tracker®, Invitrogen, Lucerne, Switzerland) in reticulocytes (Retic-COUNT positive red cells) and CD71 and CD44 expression.

### Fluorescence-activated cell sorting and flow cytometry of hematopoietic tissues

Bone marrow and spleen single-cell suspensions were immunostained for Ter119, CD71, and CD44 and CD47 (all from BD Biosciences) after blocking cellular Fc receptors with 5% rat serum and analyzed using a flow cytometer (FACSCalibur or Gallios, Becton Dickinson, Allschwil, Switzerland or Beckman Coulter, Nyon, Switzerland) and the Winmdi or Kaluza software. Hematopoietic cells labeled with CD44 and TER119 antibodies were also sorted (Aria III, 5L, Becton Dickinson), transferred onto slides, and stained with May-Grunwald solution.

### Histological analysis of hematopoietic tissues

Perfusion fixed (4% parformaldehyde, phosphate buffered saline [PBS]) and decalcified bone and spleen sections were stained with Prussian blue, Hematoxylin and eosin or, after auto-fluorescence quenching (Schnell et al. 1999), with FITC-labeled anti mouse F4/80 (Abcam, Cambridge, UK) and PE-labeled anti mouse Ter119 antibodies and 4′,6-Diamidino-2-phenylindole dihydrochloride (DAPI, Sigma, Buchs, Switzerland) to identify EIs in bone marrow. EI density and Ter119 positive cells per EI were determined (cf. legend to Fig. 5A) in 10 randomly selected, nonoverlapping fields of bone marrow sections per mouse.

Other bone marrow sections were immunostained for fibronectin (rabbit anti-human fibronectin, ICN/Cappel; goat anti-rabbit IgG, DyLight 649 labeled, Jackson ImmunoResearch Lab., Rheinfelden, Switzerland), cover slipped using a moviol-based (Calbiochem, Zug, Switzerland) embedding medium (Osborn and Weber 1982) containing 1,4-Diazobicyclo-[2.2.2]-Octan (Sigma) and hardened overnight. After image acquisition (HPX 120 C, Axioimager.Z2, Axiocam HRm CCD camera, Axiovision software version 4.5, Zeiss) average optical density levels (oDL) of single bone marrow vessels were determined (MCID Analysis 7.0) by taking into account only pixels exceeding an oDL of 45 (20% above average background value) and excluding elements with a size below 10 pixels (cf. Fig. S7B).

### Analysis of bone structure

Cortical and trabecular microarchitecture of femurs was assessed by micro-computed tomography (MicroCT40; Scanco Medical, Brüttisellen, Switzerland) scanner as described (Hildebrand and Ruegsegger 1997; Hildebrand et al. 1999; Kohler et al. 2007).

### Statistics

Results are expressed as means + SEM. Statistical significances were assessed using a two-tailed Student *t*-test for unpaired samples with Bonferroni correction and labeled as *P < 0.05, **P < 0.01, or ***P < 0.001 when compared to the respective wild-type (wt) controls or as #*P* < 0.05, ##*P* < 0.01, or ###*P* < 0.001 for comparison between β3^−/−^ and tg6 mice.

## Results

### Erythropoiesis is mildly stimulated in integrin β3^−/−^ mice

**Figure 1:**
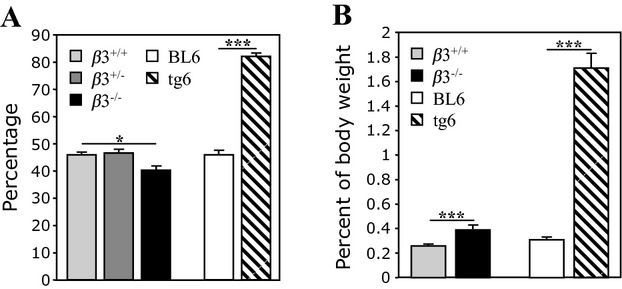
Comparison of the strength of erythropoietic stimulation in integrin β3-deficient and Epo-overexpressing (tg6) mice. (A) In homozygous β3-deficient mice the hematocrit is slightly but significantly decreased. Systemic overexpression of Epo results in extreme hematocrit values as high as 82% in line with our previous reports (Ruschitzka et al. 2000; Vogel et al. 2003; Bogdanova et al. 2007) (*n* ≥ 5). (B) Stimulation of erythropoiesis results in splenic enlargement in both genetically modified mouse lines that is, however, stronger in tg6 mice (*n* ≥ 5).

In line with our previous reports (Ruschitzka et al. 2000; Vogel et al. 2003; Bogdanova et al. 2007), average hematocrit of the tg6 mice used here was 0.83 whereas it was 12% lower in β3^−/−^ but not β3^+/−^ mice compared to wt littermates. (Fig. 1A). Accordingly in four mice of each genotype, hemoglobin concentration (g/dL: wt: 16.05 ± 0.15; β3^−/−^: 12 ± 0.7, *P* < 0.05) and red cell count (*10^6^/μL: wt: 10.82 ± 0.37; β3^−/−^: 7.7 ± 0.89, *P* < 0.01) was reduced in β3^−/−^ mice. Erythrocyte parameters such as mean corpuscular hemoglobin (pg: 15 ± 0.01; β3^−/−^: 16 ± 0.02, ns) and mean corpuscular hemoglobin concentration (g/dL: wt: 34 ± 0.03; β3^−/−^: 32 ± 1, ns) were unchanged suggesting normal hemoglobin synthesis. RBC volume was increased in β3^−/−^ mice (mean corpuscular volume (fL): wt: 44 ± 1; β3^−/−^: 49 ± 2, *P* < 0.05) with a slightly higher scatter in β3^−/−^ mice (relative distribution width: wt: 20.9 ± 0.7; β3^−/−^: 21.3 ± 1.1, ns).

The 1.5-fold and 5.5-fold splenic enlargement in β3^−/−^ and tg6 mice, compared to their respective wt controls, indicates stimulated extramedullary erythropoiesis in both genetically modified mouse lines that is, however, much stronger in the Epo-overexpressing mice (Fig. 1B). Accordingly, plasma Epo levels were unaltered in β3^−/−^ (β3^−/−^: 20.49 ± 1.45 U/L; wt: 20.35 ± 0.85 U/L, respectively, *n* = 10). Flow cytometric erythrocyte size determination and peripheral blood smears did not reveal morphological alterations in β3^−/−^ mice. Prussian blue staining of bone marrow and spleen sections did not indicate altered iron storage in β3^−/−^ mice.

### Red cell survival is normal in integrin β3^−/−^ mice

In wt mice RBC half-life was within literature values (Manodori and Kuypers 2002), namely 21.6 ± 0.67 days (*n* = 8). In integrin β3^−/−^ mice half-life was slightly (17.9 ± 2.96 days [*n* = 5]) but not significantly (*P* = 0.29) lower, maybe because of the higher data scatter in β3^−/−^ mice compared to their wt littermates (coefficient of variation 36.93% vs. 8.99%). This just marginally reduced red cell survival together with limited splenomegaly and unaltered plasma Epo levels suggest mild erythropoietic stimulation in β3^−/−^ mice.

### Mechanical properties of erythrocytes from integrin β3^−/−^ mice are normal

Mechanical properties of erythrocytes are age-dependent with young cells being more flexible and resistant to osmotic stress. Erythrocytes of tg6 mice were reported to be more flexible and having a higher osmotic resistance (Vogel et al. 2003; Bogdanova et al. 2007). Compared to wt littermates, β3^−/−^ mice showed a trend toward increased erythrocyte flexibility at higher shear forces but unchanged osmotic resistance (50% lysis of RBCs: wt: 0.5581%, β3^−/−^: 0.5545%).

### Peripheral erythrocytes of integrin β3^−/−^ mice show a higher degree of immaturity than those of tg6 mice

**Figure 2:**
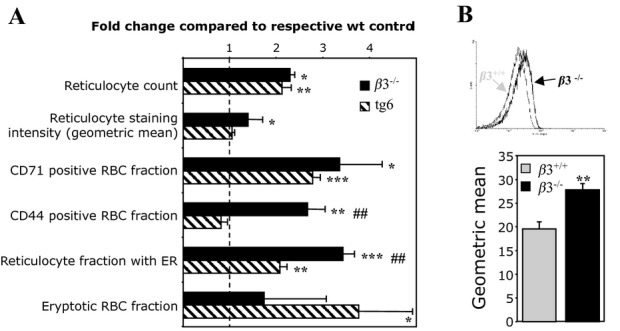
Analysis of peripheral red blood cell (RBCs) in integrin β3^−/−^ and tg6 mice. (A) This bar graph shows for both genetically modified mouse lines the fold changes of the parameters in relation to their respective wt controls (set to 1). Reticulocyte counts were significantly and similarly increased in both β3^−/−^ and tg6 mice but only β3-deficient mice showed a significantly higher reticulocyte staining intensity (*n* ≥ 6). The percentage of CD71-positive RBCs was similarly increased in β3^−/−^ and tg6 mice (*n* ≥ 6) whereas the CD44-positive fraction was significantly increased only in β3^−/−^ mice (*n* = 5). Accordingly, the ER-tracer dye positive reticulocyte faction was higher in β3^−/−^ mice (*n* = 5). Binding of annexin V to RBCs, marking the eryptotic RBC fraction, did not differ significantly between β3-deficient mice and their wt controls. In contrast, according to our previous findings (Foller et al. 2007) annexin V binding was significantly increased on tg6 erythrocytes (*n* = 6). (B) In addition, RBCs from β3^−/−^ displayed increased immunoreactivity for CD47 on their surface as evident from right-shift of the population (image) that is quantified by the geometric mean (bar graph, *n* = 4).

**Figure 3:**
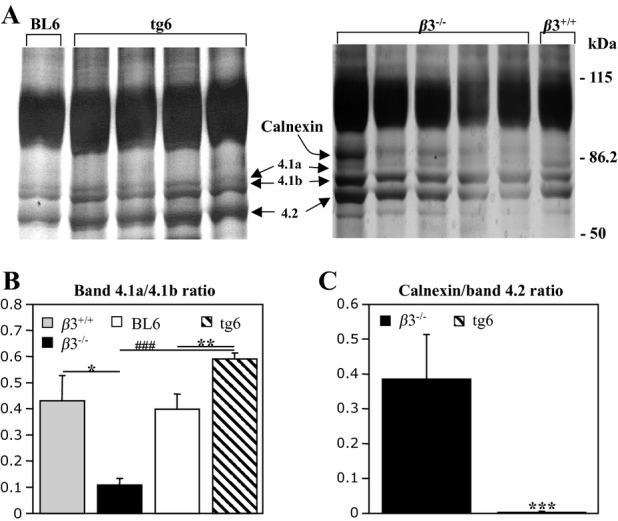
Silver-stained SDS–polyacrylamide gel electrophoresis of erythrocyte membrane proteins. (A) In β3-deficient mice an additional band was detected that was identified as calnexin using mass spectroscopy. (B) Quantification of the band 4.1a/4.1b ratio revealed in β3-deficient mice a reduction whereas in accordance with our previous study (Bogdanova et al. 2007) this ratio was increased in tg6 (*n* ≥ 5). (C) Quantification of the calnexin band intensity in relation to the band 4.2 intensity revealed practically no signal in tg6 mice whereas it was clearly detectable in β3^−/−^ mice (*n* ≥ 5).

Compared to wt, reticulocyte counts were significantly and, more surprisingly, similarly increased in β3^−/−^ and tg6 mice. Maturity of the peripheral red cells was therefore assessed by additional measurements (Figs. 2and 3).

The skewness of the intensity distribution directly impacts the geometric mean (cf. Fig. 2B) that can be used to estimate reticulocyte age distribution (Wiczling and Krzyzanski 2007, 2008). Average fluorescence intensity of Retic-COUNT®-stained reticulocytes from β3^−/−^ was significantly higher than of those of their wt littermates whereas tg6 reticulocytes had the same staining intensity as control mice. Accordingly, Brilliant-Kresyl blue-stained blood smears appeared more intense and spread in β3^−/−^ than in wt or tg6 reticulocytes. Using flow cytometry, expression of the transferrin receptor CD71 was assessed because this protein diminishes with erythroblast maturation (Kina et al. 2000). Compared to wt controls a similar and significant increase in cells positive for CD71 was observed in β3^−/−^ and tg6 mice. Next, we examined on RBCs expression of the adhesion molecule CD44 that might be more specific for late erythropoietic developmental stages (Chen et al. 2009). Compared to wt littermates as well as to tg6 mice β3^−/−^ mice had significantly more CD44 positive RBCs. In contrast, RBCs of tg6 mice showed the same CD44 expression as their wt controls. Another sign of immaturity are remnants of the ER that were considerably (+50%) more increased in β3^−/−^ than in tg6 RBCs. Red cell aging, next to other stresses, results in phosphatidylserine exposure. As reported previously (Foller et al. 2007) tg6 erythrocytes bound significantly more Annexin V whereas β3^−/−^ RBCs showed unaltered Annexin V binding (Fig. 2A). Tg6 erythrocytes express less CD47 (Bogdanova et al. 2007), a marker that declines with RBC aging (Oldenborg et al. 2000). In contrast to tg6 RBCs, β3^−/−^ red cells displayed increased CD47 expression by about 42% compared to their wt littermates (Fig. 2B).

Next, we determined the band 4.1a/4.1b ratio of RBC ghost proteins that increases with RBC aging (Inaba and Maede 1988). Figure 3A shows examples of silver-stained SDS-polyacrylamide gel electrophoresis (SDS-PAGE). For both wt strains used, the band 4.1a/4.1b ratio was within the values reported previously for mice (Inaba and Maede 1988; Bogdanova et al. 2007). In line with our previous work the band 4.1a/4.1b ratio was increased in tg6 mice compared to their wt controls (Fig. 3B), which had been interpreted as accelerated aging of the transgenic RBCs (Bogdanova et al. 2007). In contrast, in β3^−/−^ mice the band 4.1a/4.1b ratio was dramatically reduced by about 75% suggesting a reduced average age of peripheral β3^−/−^ RBCs.

Interestingly, silver-stained SDS-PAGE of RBC membrane extracts from β3^−/−^ mice showed an extra band at around 90 kD (Fig. 3A), which was identified as calnexin with a MASCOT's probability based Mowse score of 291 indicating that the probability that this protein is not present in the extra band is smaller than 10^−29^%. Sixteen peptide query matches and 9.5% sequence coverage were obtained and mass accuracy for all peptides was better than 20.5 ppm. Calnexin, an ER glycoprotein chaperone, is normally completely lost before reticulocytes are released into the circulation (Patterson et al. 2009), was absent in tg6 RBC membrane preparations (Fig. 3A and 3C) implying even abnormal immaturity of the peripheral RBCs from β3^−/−^ mice. These data suggest a reduced stability of the erythropoietic niche in β3-deficient mice.

### The latest erythroid developmental stage is reduced in bone marrow of β3^−/−^ mice

Using the flow cytometric assay developed by Liu et al. (2006), we next examined the erythroblast differentiation stages in hematopoietic tissues. Based on Ter119 and CD71 expression, bone marrow and spleen cells were classified as ProE and cells expressing high levels of Ter119.

**Figure 4:**
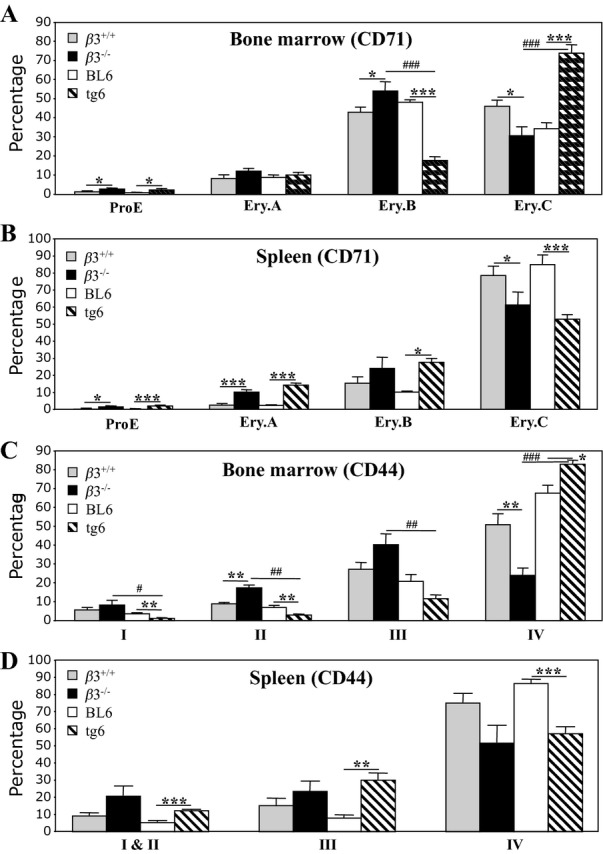
Analysis of erythroblast subsets in bone marrow and spleen. (A) In the bone marrow ProE were significantly increased to a similar extend in both β3^−/−^ and tg6 mice. Ery.A were not altered whereas Ery.B were significantly increased in β3^−/−^ mice but decreased in tg6 mice. The Ery.C population, representing orthochromatic erythroblasts and premature reticulocytes, was significantly decreased in β3^−/−^ but increased in tg6 mice (*n* = 5). (B) In the spleen no differences between β3^−/−^ and tg6 mice were found regarding the fraction of the different erythroblast subpopulations (*n* = 5). Panel (C) and (D) show the different erythroblast subpopulations after staining with FITC-conjugated anti-CD44 and PE-conjugated anti-Ter119 antibodies. (C) In line with the findings obtained with CD71 the most obvious difference between β3^−/−^ and tg6 mice was the marked decrease in the latest erythropoietic developmental stage (subpopulation IV) in β3^−/−^ but an increase in tg6 bone marrow compared to wt controls. Also the pattern of the other subpopulations was comparable to that observed with CD71. (D) As for CD71, in the spleen there was no difference between β3^−/−^ and tg6 mice regarding the erythropoietic subpopulations.

**Figure A1:**
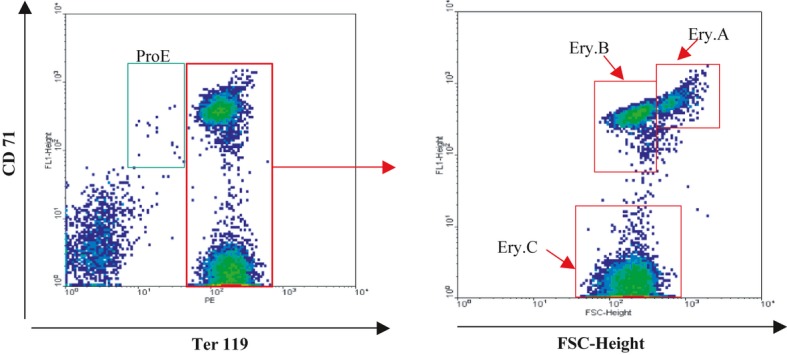
Bone marrow and spleen cells (shown here: bone marrow) were classified based on Ter119 and CD71 expression as ProE and cells expressing high levels of Ter119. The population pattern was similar for bone marrow and spleen. The whole population of Ter119_high_ cells, representing later stages of erythroblast development, was further subdivided according to its CD71 expression level and forward scatter (FSC). The resulting three subpopulations were identified as Ery.A (Ter119_high_ CD71_high_ FSC_high_), Ery.B (Ter119_high_ CD71_high_ FSC_low_) and Ery.C (Ter119_high_ CD71_low_ FSC_low_), corresponding to basophilic, polychromatic and orthochromatic erythroblasts and reticulocytes, respectively.

Splenic erythroblasts (Ter119 positive cells) were significantly increased in both genetically modified mouse lines (*n* = 8, all in%: β3^+/+^: 10.7 ± 2.56; β3^−/−^: 28.6 ± 4.85, *P* < 0.01 vs. β3^+/+^; BL6: 7.2 ± 2.65; tg6: 66.7 ± 2.74, *P* < 0.001 vs. BL6) but not in bone marrow (*n* = 8, all in%: β3^+/+^: 34.8 ± 2.31; β3^−/−^: 42 ± 3.86; BL6: 49.2 ± 2.16; tg6: 50.5 ± 2.11). The proportion of the different erythroblast subsets Ery.A, Ery.B, and Ery.C (Fig. A1) in bone marrow was clearly different between β3^−/−^ and tg6 mice. Whereas compared to their respective wt controls the percentage of ProE and Ery.A was similar in β3^−/−^ and tg6 mice, the percentage of Ery.B was increased in β3^−/−^ but lower in tg6 mice. Conversely, the percentage of the most mature erythroblast subset Ery.C that represents orthochromatic erythroblasts as well as premature reticulocytes (Liu et al. 2006) was reduced in β3^−/−^ but increased in tg6 mice (Fig. 4A). This finding is in line with more immature peripheral RBCs we found in β3^−/−^ mice. Interestingly, the high increase of Ery.C in tg6 mice compared to their wt controls might suggest delayed RBCs release from the bone marrow, which fits to our previous report that tg6 RBCs share features of young and aged erythrocytes (Bogdanova et al. 2007). In the spleen of tg6 mice the increase of ProE was much more pronounced compared to β3^−/−^ mice (11-fold vs. 4.8-fold) but no other differences between both mouse lines were found in this organ (Fig. 4B).

**Figure A2:**
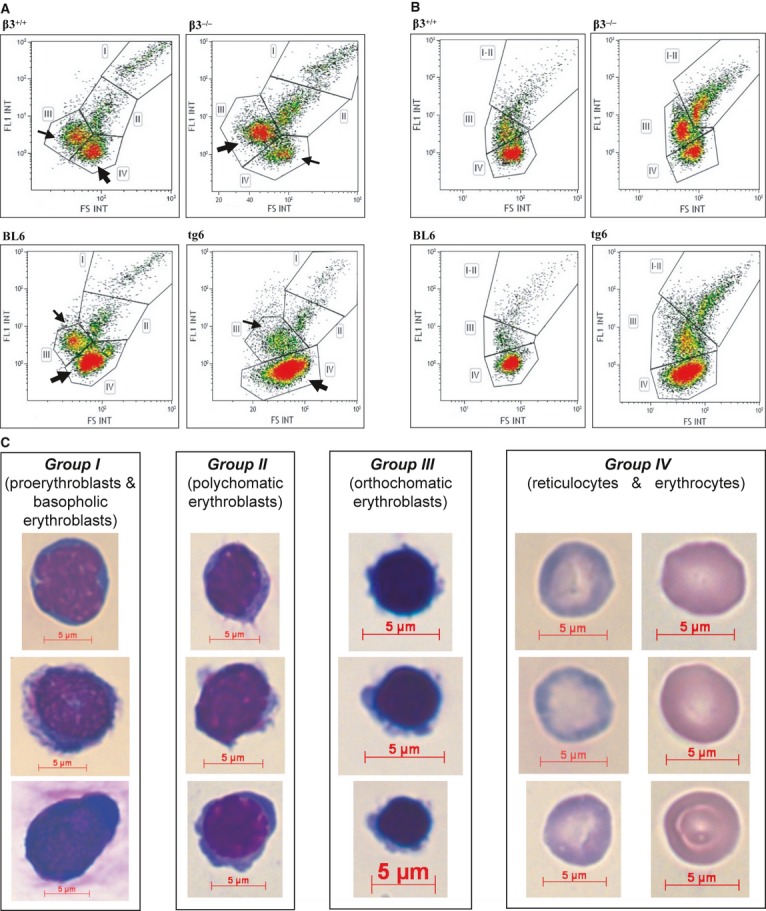
Flow cytometric analysis of bone marrow (A) and spleen cells (B) after staining with fluorescence labeled antibodies against CD44 (FL1) and TER119 (FL2) and definition of subpopulations (C). (A) In TER119 positive bone marrow cells (gated similarly as for CD71 stained cells, cf. Fig. A1 red rectangle of left panel) four distinct subpopulations could be distinguished based on CD44 staining intensity and forward scatter (FL1 INT vs. FS INT plots). These populations are labeled I, II, III and IV. Note that only in β3^−/−^ mice more cells are found in population III than IV (arrows, cf. also to Fig. 4C). In contrast, in spleen cells (B) only three subpopulations could be distinguished with the CD44 and TER119 antibodies. The population labels correspond to those in (A). (C) Cells found primarily in May-Grunwald stained cytospin preparations of the subpopulations defined in (A) and (B) after fluorescence-activated cell sorting (FACS). Subpopulation I corresponded to ProE and Ery.A, II to EryA, III to some EryB and mainly Ery.C as defined by Liu et al. (2006) and IV mainly to reticulocytes but also some erythrocytes similar to the definition from Chen et al. (2009).

A study using cultured splenic ProE and bone marrow cells obtained from mice infected with the anemia-inducing strain of Friend leukemia virus (FVA) suggests that later erythropoietic developmental stages may resolve better when stained with antibodies against CD44 (Chen et al. 2009). Therefore, we also analyzed the expression of CD44 on bone marrow and spleen cells of our mice. Compared to Chen et al. (2009) our flow cytometrically obtained pattern looked differently (Fig. A2). Consequently, we sorted the cells and identified their morphology after May-Grunwald staining (Fig. A2C). This way, in CD44- and TER119-stained mouse bone marrow cells four erythropoietic subopulations could be distinguished (Fig. A2A). Subpopulation I corresponded to ProE and Ery.A, II to EryA, III to some EryB and mainly Ery.C as defined by Liu et al. (2006) and IV mainly to reticulocytes but also some erythrocytes similar to the definition from Chen et al. (2009). In spleen cells, however, subpopulations I and II could not be separated reliably (Fig. A2B). Whereas the proportion of splenic erythropoietic stages in spleens of β3^−/−^ and tg6 mice was similarly altered compared to their respective wt control (Fig. 4D), clear differences were found in the bone marrow. As for CD71 the most striking differences between both genetically modified mouse lines were found in the last erythroid stage (IV, Fig. 4C). Compared to their nontransgenic littermates the fraction of population IV was reduced to about 40% in β3^−/−^ mice whereas in tg6 mice it was increased by about 30%. Moreover subpopulation III was increased in β3^−/−^ mice but reduced in tg6 mice although this did not reach the level of statistical significance (Fig. 4C). Note that erythropoietic stimulation resulted in an inversion of the ratio between subpopulation III (or Ery.B) and IV (or Ery.C) only in β3^−/−^ but not in tg6 (cf. Fig. 4A and C).

### β3^−/−^ EIs contain less erythroblasts

**Figure 5:**
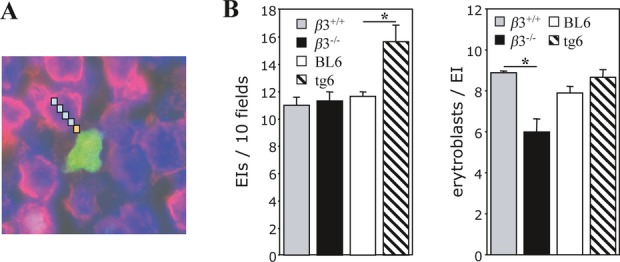
Analysis of EIs in bone marrow. (A) Representative image of a bone marrow EI with the macrophage labeled with an antibody directed against F4/80 (green) and the erythroblasts labeled with an antibody against TER119 (red). Original magnification 1000×. Only those TER119 positive cells were considered to be associated with a central macrophage that were 25% or less of the cell diameter distant from a F4/80 positive cell (four gray squares, cell diameter; yellow square, 25% of the diameter of that cell). (B) Quantification of the EI density per 10 fields of view per animal and the number of erythroblasts attached to a macrophage. Compared to wt controls there was no difference in β3^−/−^ mice regarding EI density whereas in tg6 mice it was significantly increased (left panel). In β3^−/−^ mice there was a significant reduction of cells per EI whereas tg6 mice did not show a difference compared to their wt controls regarding this parameter (right panel, *n* = 10).

Our above-mentioned flow cytometric data prompted us to next visualize bone marrow EIs with ani-F4/80 and anti-Ter119 antibodies (Fig. 5A). Compared to respective wt controls, EI density in femur marrow was unchanged in β3^−/−^ mice but significantly increased in tg6 mice. However, significantly less (−30%) erythroblasts per island were observed in β3^−/−^ whereas in tg6 mice it was comparable to wt controls (Fig. 5B).

### Fibronectin immunoreactivity of bone marrow is increased in β3^−/−^ and decreased in tg6 mice

**Figure 6:**
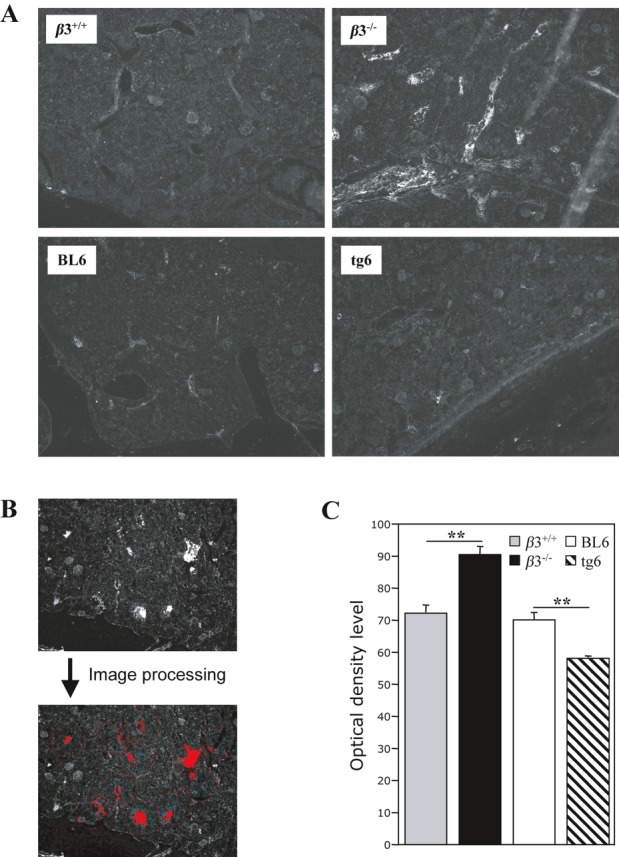
Assessment of local fibronectin expression in bone marrow. (A) Examples of immunofluorescence against fibronectin (original magnification 200×). (B) Illustration of the quantification procedure. Using an image analyzing system a threshold 20% above the average background optical density level (oDL) was defined. A circular sample tool was used to measure each single vessel in the images separately. The threshold procedure defined the pixels representing each single vessel (red). Then the average oDL of each vessel was calculated. (C) These measurements were very stable and revealed in bone marrow vessels an increased fibronectin staining intensity in β3^−/−^ mice whereas it was reduced in tg6 mice. Between both wt control groups no differences could be detected (*n* = 4).

Quantitative immunofluorescence of fibronectin staining in the bone marrow revealed quite stable measurements. Compared to their respective wt controls vessel-associated fibronectin staining intensity was significantly increased in β3^−/−^ mice but decreased in tg6 mice (Fig. 6).

### Three-dimensional bone structure of β3^−/−^ mice is not altered

**Figure 7:**
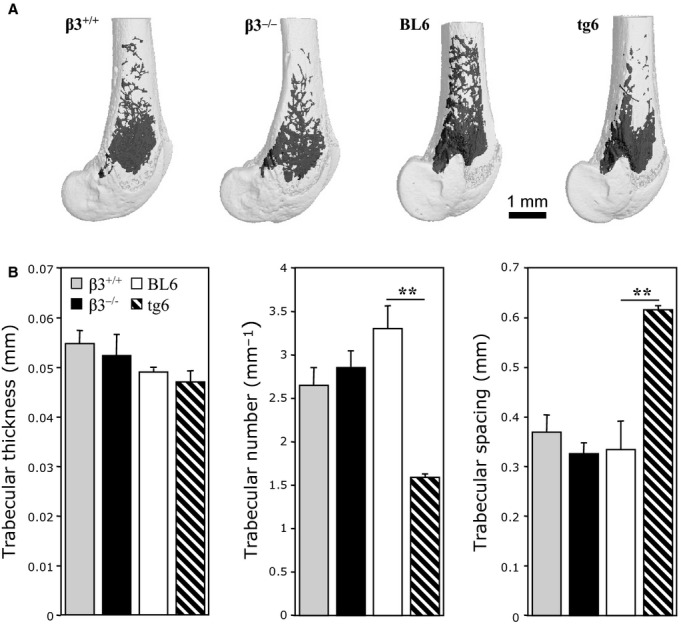
Three-dimensional analysis of trabecular bone in the distal femur. (A) Representative μCT images of the distal femur of all mouse lines investigated. Trabecular bone (black) had been detected user independently by the software as described elsewhere (Kohler et al. 2007). Note the reduced volume density of the trabecular bone in the tg6 mouse. (B) Quantification of the trabecular bone structure. Trabecular thickness was the same among all mouse lines. Tg 6 mice showed a significantly decreased trabecular number, which resulted in increased trabecular spacing. This suggests expanded space for erythropoietic cells (*n* ≥ 3).

Epo induces indirectly trabecular bone loss (Singbrant et al. 2011) to free marrow space for erythroid cell expansion. Mice lacking β3 were reported nonquantitatively to have more but dysfunctional osteoclasts resulting in increased cortical and trabecular bone mass (McHugh et al. 2000). In contrast, as shown in Figure 7, we quantified the three-dimensional structure of the femurs using microcomputed tomography imaging (Hildebrand et al. 1999). Using this technique we did not observe significant changes in the cortical bone in none of our mice. However, trabecula of tg6 bones showed reduced numbers, increased spacing but unaltered thickness. In contrast, β3^−/−^ mice displayed a slight nonsignificant trend toward reduced trabecular spacing and increased trabecular number in line with McHugh et al. (2000). Hematoxylin and eosin-stained bone sections showed no obvious differences regarding the trabecular or cortical bone structure in β3^−/−^ mice (not shown).

## Discussion

We compared a strong erythropoietic stimulus with a weak one to reveal a potential role of integrin β3 for EI integrity. Despite having in comparison to wt mice only mildly stimulated erythropoiesis (unaltered plasma Epo values, unchanged EI density in bone marrow, minor splenic enlargement, normal RBC life span) integrin β3-deficient mice exhibit peripheral RBCs of a lower maturity grade (more intense RNA staining in reticulocytes, increased CD44 and CD47 expression, increased band 4.1a/4.1b ratio) in comparison to tg6 mice with maximally stimulated erythropoiesis. Of note, β3^−/−^ mice retain calnexin in their peripheral erythrocytes indicating even abnormal immaturity. Moreover, in bone marrow from β3 deficient but not erythropoietin-overexpressing mice the late stage erythroblasts are reduced. Together with the finding of reduced numbers of erythroblasts per EI in β3-deficient mice these observations suggest that integrin β3 might play a role in the stabilization of the EIs, most likely during the late maturation stage just before reticulocyte release.

Central macrophages of EIs express αv integrin and erythroid ICAM-4 (LW glycoprotein) that is critical for EI formation (Lee et al. 2006). However, integrins, ligands for ICAMs, are functional only as heterodimers composed of one α and one β subunit. Whereas β subunits combine with different α subunits, α subunits bind only one β subunit with the exception of αv, α4, and α6 (Hynes 1992). Thus, the question remained as to the binding partners of αv integrin on the central macrophage of EIs. A hint to answer this question was the observation that the red cell ICAM-4 can bind αIIbβ3 as well as αvβ3 integrin heterodimers, both present on platelets (Hermand et al. 2003, 2004). Genetic deficiency of αIIb and β3 integrins results in a bleeding disorder known as Glanzmann thromboplasthenia (Coller et al. 1987) as in the β3 integrin-deficient mice used here (Hodivala-Dilke et al. 1999). In contrast, ICAM-4-deficient animals do not show alterations in hemostasis, hematocrit, or hemoglobin levels (Lee et al. 2006), probably because during hemostasis platelet integrins bind to extracelluar matrix molecules such as fibrinogen, fibronectin, von Willebrand factor, thrombospondin and vitronectin (Hynes 1992; Felding-Habermann and Cheresh 1993) and not ICAM-4. However, binding of RBCs to the forming thrombus requires interaction of ICAM-4 with αIIbβ3 and/or αvβ3 integrins (Hermand et al. 2003, 2004) although this has not yet been proven in vivo (Lee et al. 2006). Our present data suggest an additional function of integrin β3, namely, stabilizing EIs as heterodimerization partner of αv integrin on central macrophages. This is concluded from the notion that high erythropoietic stimulation result in reduced maturity of the reticulocyte population (Al-Huniti et al. 2005), which, however, contrasts our observation that β3 integrin-deficient mice with much weaker erythropoietic stimulation than tg6 mice exhibit abnormally immature peripheral RBCs (Figs. 3), EI's with significantly less Ter119 positive cells (Fig. 5B) and a reduced erythroid bone marrow population representing orthochromatic erythroblasts and premature reticulocytes (Fig. 4C and E).

It could be shown that different hematopoietic cell lines bind to ICAM-4, which involves α4β1 integrin and αv-family integrins but β3 and αv integrin specific antibodies failed to reduce binding of these hematopoietic cell lines to ICAM-4 (Spring et al. 2001). Maybe the stable cell lines used in the latter study do not entirely reflect the dynamic in vivo situation, an interpretation in line with the fact that the same study also shows that only six out of 12 tested hematopoietic cell lines bound ICAM-4 (Spring et al. 2001). Moreover, the same group showed later that the αv integrin is indeed involved in binding of erythroblasts to the central macrophage (Lee et al. 2006). However, the whole picture might be more complex as α4β1 integrin can in addition to ICAM-4 (Spring et al. 2001) also interact with the VCAM-1 within EIs (Sadahira et al. 1995). In this case, α4β1 integrin is expressed on erythroblasts and VCAM-1 on the central macrophage (Sadahira et al. 1995). Another hint for the importance of the integrin – ICAM-4 – interaction for RBC production suggests expression and secretion of a soluble ICAM-4 isoform by erythroblasts (Lee et al. 2003) that could compete with the membrane bound ICAM-4 for integrin counterreceptors and thus play a role for the final detachment of reticulocytes from the central macrophage. If another heterodimerization partner of αv integrin is missing, for example, β3 as we suggest, ICAM-4 dependent EI stability could be weakened and, thus, lower concentrations of soluble ICAM-4 could result in a preterm release of late stage erythroblasts or premature reticulocytes from the EI. Moreover, our data suggest that strong erythropoietic stimuli *per se* such as massive systemic Epo overexpression in tg6 mice do not trigger release of abnormally immature reticulocytes (Figs. 3, 4C, and 5).

Adhesion of the whole EI to the extracellular matrix might be important as well. Maturating EIs migrate toward the bone marrow sinusoids where young RBCs are released into the circulation (Yokoyama et al. 2003). Whereas in early developmental stages Epo plays the predominant role, interactions of differentiating RBCs with the extracellular matrix, for example, fibronectin are crucially important for the second phase of maturation (Eshghi et al. 2007) and might even provide proliferative stimuli for hematopoietic cells (Weinstein et al. 1989; -Vuillet-Gaugler et al. 1990). The fibronectin-dependent late phase of RBC maturation correlates with the migration of the EIs toward the sinusoids and both mechanisms might be regulated by interplay between integrin β1 and β3 (Danen et al. 2002). Using quantitative immunofluorescence we found in β3^−/−^ mice compared to their wt controls bone marrow fibronectin to be increased whereas in tg6 mice it was decreased (Fig. 6). In β3^−/−^ mice increased fibronectin expression could be a compensatory mechanism to enhance binding of the premature RBCs, erythroblasts, or the whole EI to the extracellular matrix to prevent the release of cells of even lower maturity into the circulation. Moreover, this compensatory mechanism could in β3^−/−^ mice enhance also the second phase of erythroid maturation that requires fibronectin interaction with α4β1 integrin (Eshghi et al. 2007), which should not be altered in β3^−/−^ mice. This interpretation fits to the significant higher fraction of Ery.B in the bone marrow of β3^−/−^ mice (Fig. 4A). In parallel, more fibronectin could facilitate migration of EIs toward the sinusoids resulting in reduced Ery.C (or group IV) population as observed in β3^−/−^ bone marrow (Fig. 4A and C). Conversely, bone marrow fibronectin staining intensity was decreased in tg6 mice. Tg6 mice need to fight against the uncontrolled RBC production. Because they cannot escape the early stage of erythroid differentiation and expansion due to transgenic Epo production reduced fibronectin expression in tg6 mice could represent a compensatory mechanism for decelerating/inhibiting the fibronectin-dependent but Epo-independent second phase of erythroid differentiation (Eshghi et al. 2007) and migration of the EIs toward the sinusoids. This would keep the young erythrocytes longer than normal in the bone marrow. Indeed, we previously demonstrated that RBCs from tg6 mice share features of young as well as senescent red cell (Bogdanova et al. 2007), which fits to this interpretation.

Bone remodeling to reduce trabecular volume density is crucial for erythropoiesis and inhibition of osteoclasts blunts the erythropoietic response to Epo (Singbrant et al. 2011). Accordingly, in tg6 mice bone marrow volume was increased on cost of trabecular number with increased trabecular spacing, an observation in line with our previous findings (Heinicke et al. 2006). In contrast, integrin β3-deficient mice were reported to have compared to wt mice impaired bone remodeling as shown by an enhanced density of tailbones that, however, contain mainly yellow marrow (Lee and Rosse 1979) and histological examples of distal femora suggesting an increased bone mass (McHugh et al. 2000). In addition, this latter study shows in β3^−/−^ mice more osteoclasts that appear dysfunctional although they retain resorptive capacity when cultured on whale dentin. Unfortunately, no quantifications of these observations are provided. Quantitative microcomputed tomography-based analysis of femora, however, did not reveal significant signs for osteosclerosis in β3^−/−^ mice. On the other hand, altered bone remodeling should affect the EI density (Singbrant et al. 2011) (that was unchanged in β3^−/−^ mice) rather than the number of erythroid progenitors per island (that was reduced in β3^−/−^ mice). Thus, it is unlikely that the probably marginally disturbed bone remodeling in β3^−/−^ mice could be causal for the observed instability of their EIs.

In conclusion, by comparing stress erythropoiesis of Epo-overexpressing mice and integrin β3^−/−^ mice we discovered a new functional role of integrin β3 for stabilization of EIs. Our findings suggest that integrin β3 is important during the very late stage of red cell production by delaying release of premature reticulocytes.
